# Epidemiology of attention-deficit/hyperactivity disorder (ADHD) in children and adolescents in Africa: a systematic review and meta-analysis

**DOI:** 10.1186/s12991-020-00271-w

**Published:** 2020-03-13

**Authors:** Getinet Ayano, Kalkidan Yohannes, Mebratu Abraha

**Affiliations:** 1 Research and Training Department, Amanuel Mental Specialized Hospital, Addis Ababa, Ethiopia; 2 School of Public Health, Curtin University, Perth, western australia Australia; 3 Department of Psychiatry, Dilla University, Dilla, Ethiopia; 4 Department of Psychiatry, Paulo’s Millennium Medical College, Addis Ababa, Ethiopia

**Keywords:** Attention-deficit/hyperactivity disorder, Epidemiology, Africa, Children, Systematic review and meta-analysis

## Abstract

**Background:**

Attention-deficit/hyperactivity disorder (ADHD) is the most common neurodevelopmental disorders in childhood and adolescence, affecting 2.2 to 17.8% of all school-aged children and adolescents. ADHD in children has been associated with a wide range of developmental deficits including limitations of learning or control of executive functions as well as global impairments of social skills. However, no review has been conducted to report the consolidated magnitude of ADHD in children and adolescents in Africa. Therefore, this systematic review and meta-analysis aimed to estimate the prevalence of ADHD in Africa.

**Methods:**

Following the PRISMA guideline, we systematically reviewed and meta-analyzed studies that investigated the prevalence of ADHD in Africa from three electronic databases (PubMed, Embase, and Scopus). We also looked at the reference lists of included studies to include other relevant studies. Subgroup and sensitivity analysis was carried out based on the study setting, tools used to measure ADHD, sex of participants, and the subtype of ADHD. Heterogeneity across the studies was evaluated using Cochran's *Q*- and the *I*^2^-test. We assessed potential publication bias using Egger's test and visual inspection of the symmetry in funnel plots.

**Results:**

In the present meta-analysis, 7452 articles were initially identified and evaluated. Of these, 12 studies that met the inclusion criteria were included in the final analysis. The pooled prevalence of ADHD in children and adolescents in Africa was 7.47% (95% CI 60–9.26). The prevalence of ADHD was apparently greater in boys (10.60%) than in girls (5.28%) with a male:female ratio of 2.01:1. In our subgroup analysis, the predominantly inattentive type (ADHD-I) was found to be the most common subtype of ADHD, followed by hyperactive–impulsive type (ADHD-HI) and the combined type (ADHD-C) with the prevalence of 2.95%, 2.77%, and 2.44% respectively. The predominantly inattentive type (ADHD-I) was the most common type of ADHD in both boys (4.05%) and girls (2.21%). The funnel plot and Egger's regression tests provided no evidence of substantial publication bias in the prevalence of ADHD.

**Conclusion:**

Our systematic review suggested a higher prevalence of ADHD (7.47%) in children and adolescents in Africa, indicating that ADHD is a serious public health problem in children and adolescents in Africa. The prevalence of ADHD was considerably greater in males than in females. The predominantly inattentive type (ADHD-I) was the most common type of ADHD in both males and females. Greater attention needs to be paid to the prevention and treatment of ADHD.

## Background

The Diagnostic Statistical Manual of fifth revision, DSM-5, defines ADHD as a neurodevelopmental disorder characterized by impairing levels of inattention, disorganization, and/or hyperactivity–impulsivity [1]. Inattention and disorganization involve failure to stay on task, seeming not to listen, and losing materials, at levels that are not consistent with age or developmental level [1, 2]. Hyperactivity–impulsivity entails overactivity, fidgeting, inability to stay seated, intruding into other people's activities, and inability to wait—symptoms that are excessive or age or developmental level [1, 2].

In childhood, ADHD frequently overlaps with other mental disorders including oppositional defiant disorder and conduct disorder [3, 4]. ADHD often persists into adulthood, with resultant impairments of social, academic, and occupational functioning [2, 5].

In DSM-5, three main nominal subtypes of ADHD are identified which are mainly based on the differential elevation of two dimensions of inattention symptoms and hyperactivity–impulsivity symptoms. The first one is the predominantly inattentive type (ADHD-I) which describes individuals with maladaptive levels of inattention, but not hyperactivity–impulsivity; the second is the predominantly hyperactive–impulsive type (ADHD-H) which is characterized by maladaptive levels of hyperactivity–impulsivity, but not inattention, and finally, the combined type (ADHD-C) which describes individuals who exhibit significant symptoms of both inattention and hyperactivity–impulsivity [2].

In a recent meta-analysis which included 179 ADHD prevalence estimates, the pooled prevalence estimate of ADHD in children and adolescents was 7.2% (95% confidence interval: 6.7 to 7.8) [1]. However, the prevalence of ADHD in adults is lower than the corresponding prevalence estimate in children and adolescents. For example, in one meta-analysis study done in 2009, the pooled prevalence estimate of adult ADHD was 2.5% (95% CI 2.1–3.1) [6]. Nevertheless, the reported prevalence showed a significant vibration across the studies ranging from 2.2 to 17.8% [7–9].

According to evidence from different worldwide scientific studies, among the three nominal subtypes of ADHD, the predominantly inattentive type (ADHD-I) is the most common subtype of ADHD, followed by combined (ADHD-C) and hyperactive–impulsive type (ADHD-HI) [4, 10–15]. ADHD-I subtype is the most prevalent subtype of ADHD in girls than in other ADHD subtypes [4, 10–15]. Epidemiological evidence also suggests that ADHD is more prevalent among males in all three subtypes [4, 11–13, 16–18].

To our knowledge, this is the first systematic and meta-analytic review that aimed to estimate the prevalence of ADHD in children and adolescents in Africa. We hypothesized that the prevalence of ADHD is notably high in children and adolescents in Africa. Therefore, the purposes of this meta-analysis are (1) estimate the overall prevalence of ADHD in Africa; (2) determine the prevalence of specific subtypes of ADHD in Africa; (3) formulate recommendations for future clinical practice and research as well as provide evidence for planners and policymakers in the area.

## Methods/design

We conducted this review following the Preferred Reporting Items for Systematic Reviews and Meta-Analyses (PRISMA) guidelines [19]. We used two strategies to identify studies—systematic search from the three electronic databases (EMBASE, PubMed, and Scopus) and hand search of the reference lists of the included studies. The following terms and keywords were applied for searching relevant studies in PubMed: (Epidemiology OR prevalence OR magnitude OR incidence) AND (ADHD OR attention deficit hyperactivity disorder OR behavioral disorder OR neurodevelopmental disorder) AND Africa. The remaining two databases (EMBASE and SCOPUS) were searched using database-specific subject headings related to the above keywords used in PubMed. No date limit was applied.

### Eligibility criteria

An article was included if it met the following criteria: first, the study was conducted in children, (2) study design was observational studies (cross-sectional and case–control study design), (3) the outcome of interest was ADHD, and (4) conducted in Africa. We excluded editorials, reviews, nonhuman subjects, and not published in the English language. We screened titles and abstracts using the prespecified inclusion and exclusion criteria before the retrieval of full-text articles for further screening. Two reviewers (KY and MA) independently performed the screening. In the second step, the two reviewers independently read the full-texts of the articles that are not excluded in the initial stage, then selected the studies that met the inclusion criteria. Any disagreements were resolved by consensus or after discussing it with a third reviewer.

### Methods for data extraction and quality assessment

Two authors (KY and MA) independently extracted the information from the included studies. We used a specific data extraction form particularly designed to extract data for this systematic review and meta-analysis. Data from the included studies were extracted to summary tables containing information on the study population, sample size, s year of publication, study design, study setting, authors, and the tools used for assessment of ADHD. Information from the included studies was extracted as depending on the assessment template prepared as recommended by PRISMA guidelines [20].

We used a modified version of the Newcastle–Ottawa Scale (NOS) to evaluate the quality of the studies included in our final analysis [21]. The NOS scale assesses quality in several domains: sample representativeness and size, comparability between participants, ascertainment of cases and statistical quality.

### Definition of outcome (ADHD)

In this study, the definition of our outcome variable (ADHD) was based on validated standard instruments designed to measure ADHD in children and adolescents. Hence, studies included in the final analysis used the following standard tools to assess ADHD: Disruptive Behavior Rating Scale (DBRS) [22], Conners-Wells Adolescent Self-Report Scale (CASS) [23], Diagnostic interview for Child and Adolescents-Revised (DICA-R) [24], Swanson, Nolan, and Pelham rating scale 4th revision (SNAP-IV-C) [25], the Vanderbilt ADHD Teacher Rating Scale (VARTRS) [13], ADHD rating scale [26], and the Diagnostic and Statistical Manual of Mental Disorders, Fourth Edition, Text Revision (DSM-IV) [27].

### Data synthesis and analysis

For the studies that reported suitable statistics, a meta-analysis with a random-effects model was conducted to calculate pooled prevalence, and 95% CIs [28]. We used comprehensive meta-analysis software version 3 to pool the estimates from the included studies. We assessed heterogeneity using *Q* and *I*^2^ statistics [28]. The *I*^2^ statistics assess the proportion of total variance across the included studies that contributed to the observed heterogeneity. In this study, the *I*^2^ statistic value of zero indicates true homogeneity, whereas the values 25, 50, and 75% were considered to represent low, medium, and high, respectively [29]. For the data identified as heterogeneous, a random-effects model was used during analysis. A leave one-out sensitivity analysis was carried out to evaluate the key studies that exert a major impact on between-study heterogeneity. In addition, to further identify the possible source of heterogeneity among the studies we conducted the subgroup and sensitivity analysis based on the gender of the participants, the study setting, the tools used to measure ADHD, and the quality of the included studies. Publication bias was assessed by funnel plot and Egger’s regression tests. Analyses with P < 0.05 were interpreted as significant.

## Results

### Identification of studies

**Fig. 1:**
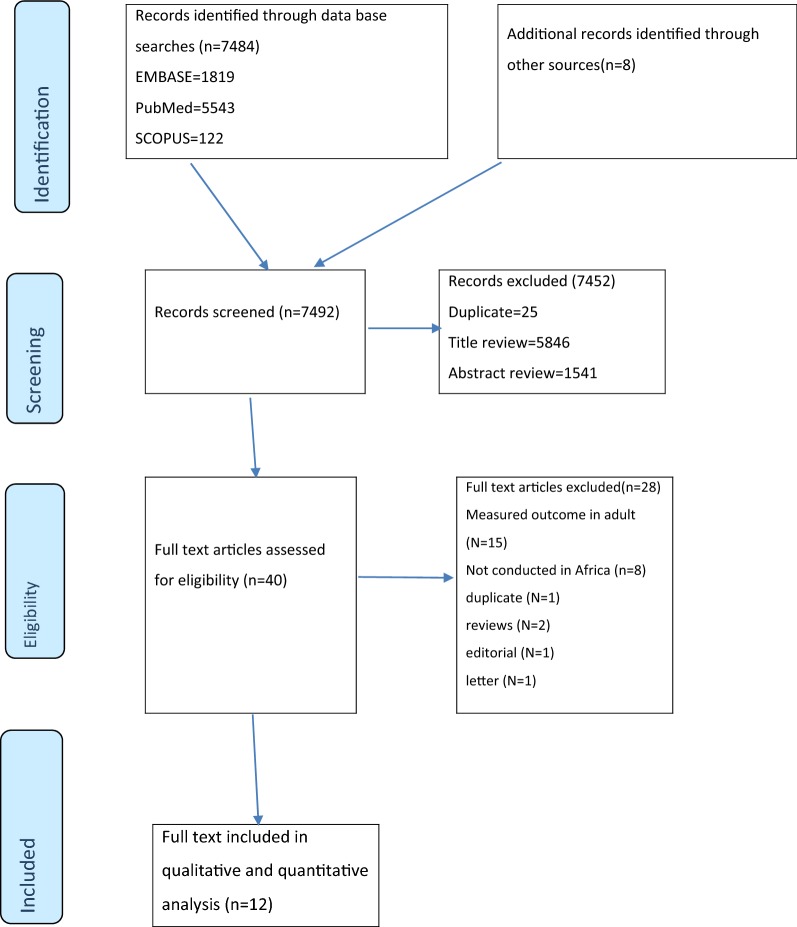
PRISMA flowchart of review search

A total of 7484 articles were identified using electronic search engines and strategies. An additional eight relevant studies were found through a manual search of the reference lists of the remaining papers. Of these, 7452 were excluded due to duplicate and during the review of abstract and titles as they did not meet the inclusion criteria (Fig. 1). 40 articles with full texts were retrieved for further screening and 28 of these were excluded.

### Characteristics of included studies

Twelve studies were included in the systematic review and meta-analysis. The characteristics of these studies are summarized in Table 1. Selected studies were conducted in Nigeria [30–32], Sudan [33], Uganda [34], Congo [35], Kenya [36, 37], Egypt [38–41], and Ethiopia [42]. Age at diagnosis of ADHD varies from 4 to 18 years. Five studies DBRS (27, 24, 25, 28, 34) to assess ADHD, one used CASS (33), one used DICA-R (35), one used SNAP-IV-C (26), one used VARTRS (23), one used ADHD rating scale (31), and two used DM-IV (29, 32). The sample size of the included studies ranged from 100 to 1477. Ten studies were good in quality, one moderate (31), and one poor (34).

**Table 1:** Characteristics of included studies

Author (year) (reference number)	Country	Sample size	Setting	Tool used	Prevalence of ADHD	NOS quality score
Wamulugwa et al. (2017) [13]	Uganda	332	Hospital based	DBRS	Overall 11.75% (*n*/*N* = 39/332) Men 14.9% (*n*/*N* = 28/188) Women 7.6 (*n*/*N* = 11/144)	8
Osman et al. (2015) [25]	Sudan	1000	Schools and community based	SNAP-IV-C	Overall 9.4% (*n*/*N* = 94/1000) Inattentive type 3.5% (*n*/*N* = 35/1000) Impulsive type 6.9% (*n*/*N* = 69/1000) Combined type %1.0 (*n*/*N* = 10/1000) Inattentive type in men 4.9% (*n*/*N* = 25/511) Inattentive type in women 1.8% (*n*/*N* = 9/489) Impulsive type in men 9.6% (*n*/*N* = 48/511) Impulsive type in women 2.3% (*n*/*N* = 21/489)	9
Adewuya et al. (2007) [22]	Nigeria	1112	Schools and community based	VARTRS	Overall 8.7% (*n*/*N* = 97/1112) Men 11% (*n*/*N* = 75/682) Women 5.1 (*n*/*N* = 22/430) Inattentive type 4.9% (*n*/*N* = 55/1112) Impulsive type 1.2% (*n*/*N* = 13/1112) Combined type % 2.6% (*n*/*N* = 29/1112) Inattentive type in men 6.2% (*n*/*N* = 42/682) Inattentive type in women 3% (*n*/*N* = 13/430) Impulsive type in men 1.6% (*n*/*N* = 11/682) Impulsive type in women 0.5% (*n*/*N* = 2/430) Combined type in men 3.2% (*n*/*N* = 22/682) Combined type in women 1.6% (*n*/*N* = 7/430)	8
Ambuabunos et al. (2011) [4]	Nigeria	1473	Community based	DBRS	Overall 7.6% (*n*/*N* = 112/1473) Men 9.4% (*n*/*N* = 74/784) Women 5.5 (*n*/*N* = 38/689) Inattentive type 3.6% (*n*/*N* = 53/1473) Impulsive type 1.6% (*n*/*N* = 24/1473) Combined type % 2.4% (*n*/*N* = 35/1473) Inattentive type in men 4.5% (*n/N* = 35/784) Inattentive type in women 2.6% (*n/N* = 18/689) Impulsive type in men 1.9% (*n*/*N* = 15/784) Impulsive type in women 1.3% (*n*/*N* = 9/689) Combined type in men 3.1% (*n*/*N* = 24/784) Combined type in women 1.5% (*n*/*N* = 11/689)	9
Ofovwe et al. (2009) [5]	Nigeria	1384	Community based	DBRS	Overall 8% (*n*/*N* = 111/1384) Men 9.5% (*n*/*N* = 69/732) Women 6.4% (*n*/*N* = 42/652) Inattentive type 2.7% (*n*/*N* = 38/1384) Impulsive type 3% (*n*/*N* = 41/1384) Combined type % 2.5% (*n*/*N* = 35/1384) Inattentive type in men 3% (*n*/*N* = 22/732) Inattentive type in women 2.4% (*n*/*N* = 16/652) Impulsive type in men 3.2% (*n*/*N* = 23/732) Impulsive type in women 2.7% (*n*/*N* = 18/652) Combined type in men 3.6% (*n*/*N* = 26/732) Combined type in women 1.4% (*n*/*N* = 9/652)	9
Kashala et al. (2005) [26]	Congo	1187	Community based	DBRS	Overall 6% (*n*/*N* = 70/1187) Men 7.1% (*n*/*N* = 38/534) Women 4.9% (*n*/*N* = 32/653)	8
Wamithi et al. (2015) [27]	Kenya	240	Institution	DSM-IV	Overall 6.3 (*n*/*N* = 15/240) Inattentive type 1.3% (*n*/*N* = 3/240) Impulsive type 2.9% (*n*/*N* = 7/240) Combined type % 2.1% (*n*/*N* = 5/240)	8
Ashenafi et al. (2001) [33]	Ethiopia	1477	Community based	DICA-R	Overall 1.49% (*n*/*N* = 22/1477)	8
Awadalla et al. (2016) [29]	Egypt	873	School based	ADHD Rating Scale	Overall 12.60 (*n*/*N* = 110/873)	7
Farahat et al. (2014) [30]	Egypt	1362	Community based	DSM-IV	Overall 6.9% (*n*/*N* = 94/1362) Men 10.9% (*n*/*N* = 73/667) Women 3% (*n*/*N* = 21/695) Inattentive type 2.6% (*n*/*N* = 36/1362) Impulsive type 1.3% (*n*/*N* = 18/1362) Combined type % 2.9% (*n*/*N* = 40/1362) Inattentive type in men 3.4% (*n*/*N* = 23/667) Inattentive type in women 1.8% (*n*/*N* = 13/695) Impulsive type in men 2.2% (*n*/*N* = 15/667) Impulsive type in women 0.4% (*n*/*N* = 3/695) Combined type in men 5.2% (*n*/*N* = 35/667) Combined type in women 0.7% (*n*/*N* = 5/695)	9
Bishry et al. (2014) [13]	Egypt	925	Community based	CASS	Overall 9.4% (*n*/*N* = 87/925) Men 13.8% (*n*/*N* = 58/421) Women 5.8% (*n*/*N* = 29/504) Inattentive type 1.2% (*n*/*N* = 11/925) Impulsive type 4.4% (*n*/*N* = 41/925) Combined type % 2.5% (*n*/*N* = 23/925) Inattentive type in men 2.3% (*n*/*N* = 10/421) Inattentive type in women 0.2% (*n*/*N* = 1/504) Impulsive type in men 8.8% (*n*/*N* = 37/421) Impulsive type in women 1.4% (*n*/*N* = 7/504) Combined type in men 2.9% (*n*/*N* = 12/421) Combined type in women 2.1% (*n*/*N* = 11/504)	9
Yahia et al. (2014) [32]	Egypt	100	Institution based	DBRS	Overall 8% (*n*/*N* = 8/100) Inattentive type 4% (*n*/*N* = 4/100) Impulsive type 6% (*n*/*N* = 6/100) Combined type % 2% (*n*/*N* = 2/100)	5

*DBRS* Disruptive Behavior Rating Scale, *VARTRS* The Vanderbilt ADHD Teacher Rating Scale, *SNAP-IV-C* Swanson, Nolan, and Pelham rating scale 4th revision, *CASS* Conners-Wells Adolescent Self-Report Scale, *DICA-R* Diagnostic interview for Child and Adolescents-Revised, *VARTRS* Vanderbilt ADHD Teacher Rating Scale, *DSM-IV* Diagnostic and Statistical Manual of Mental Disorders, Fourth Edition

### Quality assessment

The quality of the studies was assessed using the Newcastle–Ottawa Scale with some modifications. Ten studies were good in quality (23–30, 32, 33, 35), one moderate (31), and one poor (34) (see Table 1).

### The prevalence of ADHD in children (meta-analysis)

**Fig. 2:**
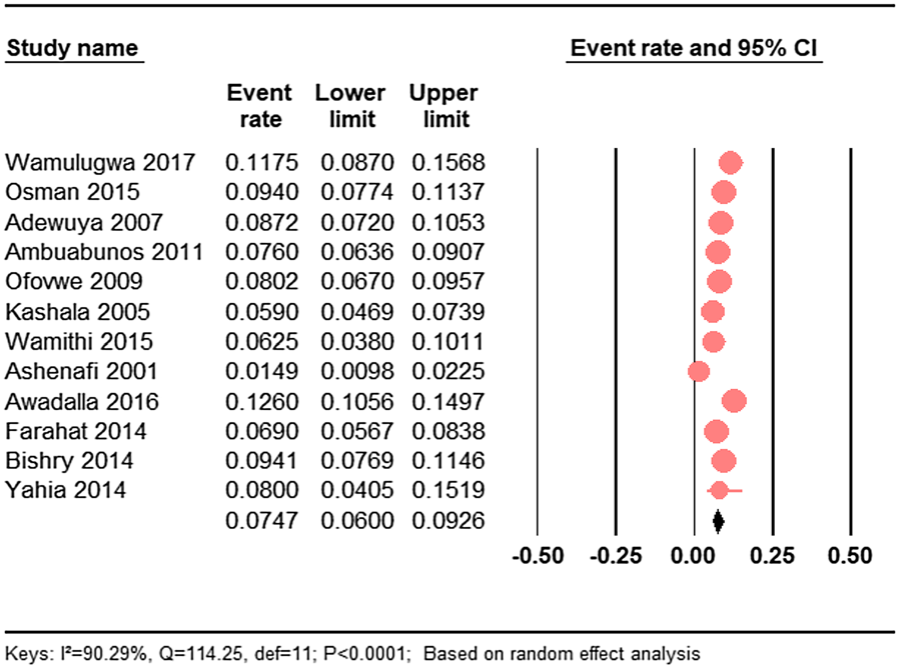
The forest plot of the prevalence of ADHD in children and adolescents in Africa: a meta-analysis

A meta-analysis of 12 prevalence studies (nine population-based prevalence studies and three clinical samples) performed in Africa between 2005 and 2017, and revealed that the pooled prevalence of ADHD in children and adolescents was 7.47% (95% CI 6.00–9.26) (Fig. 2). The result showed a significant heterogeneity across all studies (*I*^2^ = 90.29%; *Q* = 114.25, *df* = 11, *P* < 0.001). The pooled prevalence was based on the random effect model due to the observed heterogeneity across the studies.

### Sensitivity and subgroup analysis

Given a considerable heterogeneity across the included studies in the meta-analysis, we performed a sensitivity analysis to better understand the source of heterogeneity (Table 2). There was no considerable difference in the subgroups based on study setting or locations as well as nominal types of ADHD and tools used to measure ADHD. However, we identified a significant heterogeneity by gender for overall as well as subtypes of ADHD.

**Table 2:** Subgroup analysis of all studies based on study setting, sex of the participants, tools used measure ADHD, subtypes of ADHD, and study quality

Subgroups	Studies, *n*	Prevalence (%)	95% CI	Heterogeneity between groups (*P* value)
Setting				
Community based	9	7.19	5.59–9.19	0.439
Hospital based	3	8.74	5.66–13.27	
Gender				
Males	7	10.60	9.05–12.38	< 0.001
Females	7	5.20	4.38–6.36	
Subtype of ADHD				
Inattentive	8	2.95	2.23–3.89	0.539
Hyperactive	8	2.77	1.67–4.57	
Combined	8	2.44	2.01–2.96	
Inattentive type by sex				
Males	6	4.05	3.11–5.27	0.004
Females	6	2.21	1.61–3.03	
Hyperactive type by sex				
Males	6	3.61	1.88–6.82	0.062
Females	6	1.50	0.78–2.87	
Combined type by sex				
Males	5	3.63	2.87–5.87	< 0.001
Females	5	1.52	1.11–2.08	
Quality of studies				
High	10	7.04	5.61–8.79	0.041
Moderate and poor	2	11.25	7.61–16.33	
Tools used to measure ADHD				
DSM	2	6.81	5.67–8.15	0.476
Other	10	7.62	5.92–9.75	

### Gender difference in the prevalence of ADHD in children

A meta-analysis of seven studies that reported data on each gender showed that the prevalence of ADHD in males was substantially higher than the magnitude of AGHD in females. The pooled prevalence of ADHD was found to be 10.60% (95% CI 9.05–12.38) in males and it was 5.28% (95% CI 4.38–6.36%) in females. The difference between the groups was statistically significant (see Table 2).

### The prevalence of subtypes of ADHD in children

We also performed subgroup analysis-based subtypes of ADHD. In our meta-analysis, among the three nominal subtypes of ADHD, the predominantly inattentive type (ADHD-I) was found to be the most common subtype of ADHD, followed by hyperactive–impulsive type (ADHD-HI) and combined (ADHD-C) with the prevalence of 2.95%, 2.77%, and 2.44%, respectively, although the difference across the groups was not statistically significant (see Table 2).

### Gender difference in the prevalence of subtypes of ADHD in children

ADHD is more prevalent among males in all the three subtypes of ADHD, such as the predominantly inattentive type (ADHD-I), hyperactive–impulsive type (ADHD-HI), and combined (ADHD-C) with the prevalence of 4.05%, 3.61%, and 3.62%, respectively, as compared to the corresponding prevalence in females of 2.21%, 1.5%, and 1.52%. The difference was statistically significant for inattentive and combined type but not for hyperactive type (see Table 2).

ADHD-I subtype is the most prevalent subtype of ADHD in both males and females as compared to the other ADHD subtypes followed by ADHD-HI, and ADHD-C (see Table 2).

### The prevalence of subtypes of ADHD in children by study setting

The prevalence of ADHD was found to be 7.19% (95% CI 5.59–9.19) for studies conducted in a community-based setting and it was 8.74% (95% CI 5.66–13.27%) for studies conducted in hospital-based setting (see Table 2).

### Subgroup analysis by the tools used to measure ADHD

The prevalence of ADHD was found to be slightly lower when measured using DSM (6.81%) as compared to other instruments (7.62%), although the observed difference across the tools used to measure ADHD was not statistically significant (*P* = 476) (see Table 2).

### Subgroup analysis by the quality of studies

When restricting the analysis to only 11 high-quality studies (Newcastle–Ottawa Scale, ≥ 8), the magnitude of ADHD was 7.04% (95% CI 5.61–8.79) and was 11.25% (95% CI 7.61–16.33) for both moderate and poor-quality studies combined (see Table 2).

### Publication bias

**Fig. 3:**
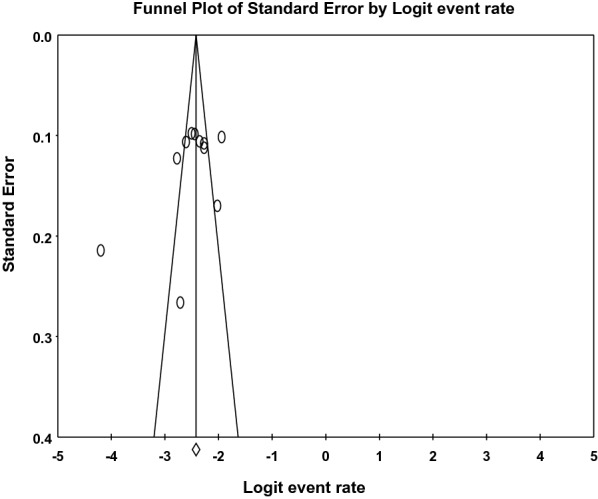
The funnel plot for assessing publication bias

Qualitatively (visual inspection), the funnel plot was symmetric and quantitatively, Egger’s regression tests provided no evidence of substantial publication bias for the prevalence of ADHD in children and adolescents in Africa (*B* = − 3.64, SE = 2.84, *P* = 0.229) (see Fig. 3).

## Discussion

### Main findings

This study is, to our knowledge, the first systematic review and meta-analysis on the prevalence of ADHD in children in Africa. We compiled data from 11,465 children selected from both community and clinical settings and estimated the prevalence of ADHD to be 7.47% (95% CI 6.00–9.26). We observed a wide variation in the prevalence of ADHD across the countries ranging from 1.49 in Ethiopia to 11.75% in Uganda. The observed substantial variation in the prevalence of ADHD across the countries could be due to the methodological differences, the age of the child, perinatal complications, the instruments used to measure ADHD, sex of the participants, culture, and the sociodemographic status of the family as well as other comorbid mental and neurologic conditions.

The finding of the current meta-analysis (7.47%) was consistent with the 9.2% worldwide pooled prevalence estimate of ADHD reported by Ramtekkar et al. [43] in 2010 by including studies conducted using diagnostic instruments such as the Diagnostic and Statistical Manual of Mental Disorders (DSM) and the International Classification of Disease (ICD) criteria’s. Likewise, our finding was also consistent with the 7.1% worldwide prevalence estimate of a recent meta-analysis conducted in 2015 by Thomas et al. [44].

However, our pooled prevalence estimates of ADHD in children and adolescents in Africa exceed the 5.3% worldwide prevalence estimate of ADHD reported in 2007 by Polanczyk et al. [45]. The possible reason for a greater estimate of ADHD in Africa might be due to the variations in the characteristics of the studies included in the meta-analysis. We included studies conducted using any type of instrument as compared to Polanczyk et al. [45] which included studies conducted only using diagnostic instruments (prevalence reported according to DSM or the ICD criteria). In fact, the observed variation across the studies that used DSM/ICD and other instruments to measure ADHD was not statistically significant in our sensitivity analysis (*P* = 476). Moreover, the study population differed in a number of mental health-related and other environmental factors such as maternal substance used during and before pregnancy, the age of the child, perinatal complications, culture, other psychological and social factors as well as the sociodemographic status of the family and other comorbid mental and neurologic conditions among the study participants that are consistently associated with increased rates of ADHD in children and adolescent might contribute to the difference in the observed variation.

As expected, our stratified analysis showed that the estimate of ADHD in males (10.60%) was considerably higher than the estimates in females (5.28%) with a male:female ratio of 2.01:1. This ratio was lower than those reported by Ramtekkar et al. [43] which provided a greater prevalence of ADHD in males than females with a male:female ratio of 2.28:1. The lower male:female ratio in our study could be due to the prevalence estimates for male (10.60%) in our review are slightly lower than those reported in Ramtekkar et al. [43] (15.7%), but the estimate of females (5.28%) was in line with those reported in Ramtekkar et al. [43] (7.5%). These findings suggest males might be underdiagnosed in Africa.

Consistent with the previous meta-analytic study, the predominantly inattentive type (ADHD-I) is the most common subtype of ADHD in our meta-analysis [7]. The consistent nature of this subtype of ADHD may contributed to the greater prevalence [46]. Complementing this, evidence suggests that inattentive symptoms of ADHD remain relatively consistent over time [46, 47] whereas the other subtypes of ADHD are such as hyperactive–impulsive symptoms are developmentally sensitive and tend to decline over time (while the feeling of restlessness can persist) [46, 47]. Contrary to the findings of worldwide prevalence estimate of the meta-analysis conducted in 2015 by Skounti et al. [7] where ADHD-C was the second most type of ADHD, in our study the second most prevalent type of ADHD was the hyperactive–impulsive type (ADHD-HI), followed by combined (ADHD-C). The possible reason for the difference might the methodological difference across the studies included in the meta-analysis as well as the variations in other factors contributing to ADHD in children and adolescents.

Moreover, the current meta-analysis ADHD is more prevalent among males in all three subtypes including the predominantly inattentive type (ADHD-I), hyperactive–impulsive type (ADHD-HI), and combined (ADHD-C). This is consistent with other study findings that ADHD is more prevalent among males in all three subtypes [4, 7, 11–13, 16–18]. In agreement with previous studies of the three types of ADHD, the prevalence of the predominantly inattentive type (ADHD-I) was greater in both males and females [4, 10–15].

### Difference between studies

In the present meta-analysis, the variant among the 12 studies included in the final analysis led to a high level of heterogeneity. The methodological difference, sample size, the setting, the tools used, and the study populations differed on a number of characteristics, which may contributed to the variance in prevalence rates of ADHD in children and adolescents in Africa. For the purpose of further investigating the potential source of heterogeneity in the analysis of the prevalence rates of ADHD in children and adolescents in Africa, extensive sensitivity analysis based on the sex of participants, the tools used, the setting, and the quality of studies was done. This analysis revealed that the major causes for the variation across the studies were the sex of the participants and the subtypes of ADHD. Moreover, to make the results of our meta-analysis meaningful, we employed a random-effects model where summary effect estimates are more conservative than fixed-effects summaries in epidemiologic meta-analysis.

### Strength and limitations

The current systematic review and meta-analysis has several strengths: first, to minimize the possible reviewer bias, data extraction and quality assessment were performed by two independent reviewers and we used a predefined search strategy to identify the possible studies to include in the study; second, to identify the small study effect and the risk of heterogeneity, we performed sensitivity and subgroup analysis; and third, we evaluated the quality of the included studies and the result from the assessment of the study quality indicated that the methodological quality was generally good. Nevertheless, we identified considerable heterogeneity among the studies which we considered as limitations of the current study.

## Conclusion

In summary, results from this systematic review and meta-analysis suggest that (1) the prevalence of ADHD (7.47%) was high; (2) the prevalence of ADHD was considerably greater in males than females with a male:female ratio of 2.01:1; (3) the predominantly inattentive type (ADHD-I) is the most common subtype of ADHD followed by ADHD-impulsive type (ADHD-HI) and combined (ADHD-C); (4) ADHD is more prevalent among males in all three subtypes; (5) among the three types of ADHD, the prevalence of the predominantly inattentive type (ADHD-I) was greater in both males and females; (6) future studies should assess the possible reasons for gender difference in epidemiology of ADHD as well as the low male:female ratio in Africa as compared to worldwide findings. Finally, more attention should be paid to the prevention and treatment of ADHD in children and adolescents in Africa.

## Data Availability

All data generated or analyzed in this study are included in this article.

## Abbreviations

ADHD: Attention-deficit/hyperactivity disorder
DBRS: Disruptive Behavior Rating Scale
CASS: Conners-Wells Adolescent Self-Report Scale
DICA-R: Diagnostic interview for Child and Adolescents-Revised
SNAP-IV-C: Swanson, Nolan, and Pelham rating scale 4th revision
VARTRS: Vanderbilt ADHD Teacher Rating Scale
DSM-IV: Diagnostic and Statistical Manual of Mental Disorders, Fourth Edition
